# Optimal timing for assessing post-intensive care syndrome in clinical research: a scoping review and expert survey

**DOI:** 10.1186/s40560-025-00817-8

**Published:** 2025-08-18

**Authors:** Kohei Tanaka, Nobuto Nakanishi, Keibun Liu, Kyohei Miyamoto, Akira Kawauchi, Masatsugu Okamura, Sho Katayama, Yuki Iida, Yusuke Kawai, Junji Hatakeyama, Toru Hifumi, Takeshi Unoki, Daisuke Kawakami, Fumimasa Amaya, Kengo Obata, Hidenori Sumita, Tomoyuki Morisawa, Norihiko Tsuboi, Ryo Kozu, Shunsuke Takaki, Junpei Haruna, Kohei Ota, Yoshihisa Fujinami, Nobuyuki Nosaka, Kasumi Shirasaki, Shigeaki Inoue, Osamu Nishida, Kensuke Nakamura

**Affiliations:** 1 Department of Rehabilitation Medicine, Osaka International Medical & Science Center, 2-6-40 Karasugatsuji, Tennoji-Ku, Osaka, 543-8922 Japan; 2 Division of Disaster and Emergency Medicine, Department of Surgery Related, Kobe University Graduate School of Medicine, 7-5-2 Kusunoki-Cho, Chuo-Ku, Kobe, 650-0017 Japan; 3 Non-Profit Organization ICU Collaboration Network (ICON), 2-15-13 Hongo, Bunkyo-ku, Tokyo 113-0033 Japan; 4 Department of Emergency and Critical Care Medicine, Wakayama Medical University, 811-1 Kimiidera, Wakayama, Wakayama 641-8509 Japan; 5 Department of Critical Care and Emergency Medicine, Japanese Red Cross Maebashi Hospital, 389-1 Asakura-Machi, Maebashi, Gunma 371-0811 Japan; 6 Berlin Institute of Health Center for Regenerative Therapies (BCRT), Charité – Universitätsmedizin Berlin, Augustenburger Platz 1, 13353 Berlin, Germany; 7 Department of Rehabilitation Medicine, Okayama University Hospital, 2-5-1 Shikata, Kitaku, Okayama 700-8558 Japan; 8 Department of Physical Therapy, School of Health Sciences, Aichi Shukutoku University, 2-9 Katahira, Nagakute, 480-1197 Japan; 9 Department of Nursing, Fujita Health University Hospital, 1-98 Dengakugakubo, Kutsukake-Cho, Toyoake, Aichi 470-1192 Japan; 10 Department of Emergency and Critical Care Medicine, Osaka Medical and Pharmaceutical University, 2-7 Daigaku-Machi, Takatsuki, Osaka 569-8686 Japan; 11 Department of Emergency and Critical Care Medicine, St. Luke’s International Hospital, 9-1 Akashi-Cho, Chuo-Ku, Tokyo 104-8560 Japan; 12 Department of Acute and Critical Care Nursing, School of Nursing, Sapporo City University, Kita 11 Nishi 13, Chuo-Ku, Sapporo, 060-0011 Japan; 13 Department of Intensive Care Medicine, Iizuka Hospital, 3-83 Yoshio-Machi, Iizuka, Fukuoka 820-8505 Japan; 14 Department of Pain Management and Palliative Care Medicine, Kyoto Prefectural University of Medicine, 465 Kajii-Cho, Kawaramachi-Hirokoji, Kamigyo-Ku, Kyoto, 602-8566 Japan; 15 Department of Rehabilitation, Japanese Red Cross Okayama Hospital, 2-1-1 Aoe, Kita-Ward, Okayama, 700-8607 Japan; 16 Clinic Sumita, 305-12 Minamiyamashinden, Ina-Cho, Toyokawa, Aichi 441-0105 Japan; 17 Department of Rehabilitation, Kobe Rehabilitation Hospital, 1-18 Shiawasenomura, Kita-Ku, Kobe, 651-1106 Japan; 18 Department of Critical Care and Anesthesia, National Center for Child Health and Development, 2-10-1 Okura, Setagaya, Tokyo 157-8535 Japan; 19 Department of Rehabilitation Medicine, Nagasaki University Hospital, 1-7-1 Sakamoto, Nagasaki, 852-8501 Japan; 20 Department of Critical Care Medicine, Yokohama City University Hospital, 3-9 Fukuura, Kanazawa-Ku, Yokohama, Kanagawa 236-0004 Japan; 21 Department of Intensive Care Medicine, School of Medicine, Sapporo Medical University, South-1, West-16, Chuo-Ku, Sapporo, Hokkaido 060-8543 Japan; 22 Department of Emergency and Critical Care Medicine, Graduate School of Biomedical and Health Sciences, Hiroshima University, 1-2-3 Kasumi, Minami-Ku, Hiroshima, 734-8551 Japan; 23 Department of Emergency Medicine, Kakogawa Central City Hospital, 439 Kakogawacho Honmachi, Kakogawa-City, Hyogo 675-8611 Japan; 24 Department of Intensive Care Medicine, Graduate School of Medical and Dental Sciences, Institute of Science Tokyo, 1-5-45 Yushima, Bunkyo-Ku, Tokyo 113-8510 Japan; 25 Department of Anesthesiology and Critical Care Medicine, School of Medicine, Fujita Health University, 1-98 Dengakugakubo, Kutsukake-Cho, Toyoake, Aichi 470-1192 Japan

**Keywords:** Critical illness, Intensive care unit, Physical function, Cognitive function, Mental health, Family

## Abstract

**Background:**

Since the concept of post-intensive care syndrome (PICS) was proposed, numerous studies have assessed patients and their family members. However, a wide range of assessment timings has been employed across previous studies. This study aimed to clarify how assessment timings have been implemented in existing PICS research through a scoping review, and to explore expert opinions on optimal assessment timing via an online survey.

**Methods:**

We conducted a scoping review of studies assessing PICS-related outcomes, including physical, cognitive, and psychological impairments, as well as PICS in family members. Studies were retrieved from MEDLINE, CENTRAL, and CINAHL, and screened by two independent pairs of reviewers. Eligible studies were published between January 2014 and December 2022. Studies lacking a clear description of assessment timing were excluded. We analyzed the reference point used to determine assessment schedules, the assessment time points, and their frequency. Additionally, an online questionnaire was administered to 23 members of the Japanese Society of Intensive Care Medicine PICS committee and working group members to collect expert opinions on these three aspects for clinical research.

**Results:**

A total of 657 studies were included. In prior studies, hospital discharge was the most commonly used reference point for determining assessment schedule (240 studies, 40%). However, ICU discharge was identified by experts as the ideal reference point (16 votes, 47%). The most frequently used assessment time points were 3 months (262, 23%), 6 months (212, 19%), and 12 months (206, 18%) post-discharge. Experts most commonly selected the period between 6 and 12 months as the optimal time point for assessment. While single assessments were most common in previous studies (337, 51%), experts considered three assessments to be ideal (12, 44%).

**Conclusions:**

This study revealed notable discrepancies between the assessment timing reported in previous studies and the opinions of experts regarding optimal timing. Standardization of assessment timing in PICS research is warranted to enhance methodological consistency and comparability.

**Supplementary Information:**

The online version contains supplementary material available at 10.1186/s40560-025-00817-8.

## Background

Recent developments in intensive care medicine have significantly reduced the mortality rates of critically ill patients [1, 2]. Post-intensive care syndrome (PICS) refers to the long-lasting physical, cognitive and psychological impairments experienced by patients after surviving intensive care, which contribute to a diminished quality of life (QOL) [3]. The proposal of PICS has increased the attention in the field of intensive care medicine toward improving the QOL of ICU survivors.

Numerous studies have been conducted in response to the growing interest in PICS. It has been reported that more than half of ICU survivors experience one or more symptoms of PICS after hospital discharge [4]. Approximately 90% of acute respiratory distress syndrome (ARDS) survivors reported persistent declines in physical function during a 5-year follow-up period [5]. Cognitive dysfunction affected nearly 50% of ICU survivors during a 1-year follow-up [6]. Furthermore, symptoms of anxiety, depression, and post-traumatic stress disorder (PTSD) persisted over a 2-year follow-up period, with prevalence rates ranging from 20 to 40% [7].

As mentioned, longitudinal studies provide important insights into the long-term outcomes of PICS. However, variations in assessment tools and timing across studies complicate the integration and comparison of study results. Our group previously conducted a scoping review using a modified Delphi method to recommend instruments for assessing PICS [8]. Other studies have also explored the optimal instruments for PICS assessments [9, 10]. With respect to the timing of assessments, the Society of Critical Care Medicine recommends screening PICS-related impairments within 2 to 4 weeks following hospital discharge, with continued reassessments throughout the recovery process [11]. However, the timing of continued reassessments has not been clarified. It is important to identify the reference point used to determine assessment schedules, the assessment time points, and their frequency. Standardizing the timing of assessment to reduce methodological heterogeneity represents a critical step toward advancing future research on PICS.

Reviewing the assessment timing used in previous studies is essential for understanding previous research trends. However, the optimal timing of assessment may vary depending on the specific context and objectives of each study. Therefore, expert opinions are required for the interpretation of the scoping review findings. Summarizing the perspectives of PICS experts will provide further valuable insights into determining the optimal assessment timing for future PICS studies. The two objectives of this study were: (1) to investigate the assessment timing in previous PICS studies through a scoping review, and (2) to summarize expert opinions on the optimal timing through an expert survey.

## Methods

### Study design

We utilized data from a previously conducted study that employed a scoping review and a modified Delphi method to recommend instruments for PICS assessment [8]. The original study was registered as a clinical trial (UMIN Clinical Trials Registry: 000049634). As this is a post hoc analysis of a scoping review based on publicly available data from databases, approval from an ethics committee was not required.

### Studies from the original scoping review

The original scoping review screened studies evaluating PICS-related outcomes including physical, cognitive, and mental health, QOL, activities of daily living, PICS-family (PICS-F) domains, and other domains in patients discharged from the ICU. The following databases were searched: Medical Literature Analysis and Retrieval System Online (MEDLINE) via PubMed, the Cochrane Central Register of Controlled Trials (CENTRAL), and the Cumulative Index to Nursing and Allied Health Literature (CINAHL) [8]. The search covered publication from 2014 to December 2022. Eligible studies included observational studies and randomized controlled trials enrolling adult ICU survivors (≥ 18 years old) and/or their family members, with PICS assessments conducted at hospital discharge or later. Exclusion criteria included review articles, study protocols, trial registries, case reports, conference abstracts, and non-English-language publications. Detailed search strategies are presented in Additional File 1. After duplicate removal, studies were screened in two stages using Rayyan, according to predefined inclusion and exclusion criteria. Screening was independently conducted by two pairs of reviewers (K.T., A.K., M.O., S.K.). Titles and abstracts were screened in the first stage, and full texts in the second. Disagreements were resolved through discussion or, if needed, by a third reviewer (N.Nakanishi).

### Additional screening and outcome assessment

An additional screening was performed to include only studies that clearly specified the timing of PICS assessments. Studies lacking a representative assessment timing or exhibiting considerable variability across patients (e.g., several months or years) were excluded. This screening was conducted by a single reviewer (K.T.). The following outcomes were extracted: (1) reference point used to determine assessment schedules, (2) assessment time point, and (3) frequency of assessment. Furthermore, we analyzed the assessment interval between each assessment time point. Assessment time point and frequency of assessment were classified into each PICS domain including physical, cognitive, mental health, QOL, and PICS-F. For studies assessing multiple domains, data were counted separately under each corresponding domain.

The reference point was defined as the day from which assessment time point was measured. For instance, the phrase “1 month after ICU discharge” differs from “1 month after hospital discharge.” Reference points were categorized into ICU admission, ICU discharge, hospital discharge, and others (e.g., death, intervention start, ventilator weaning, treatment withdrawal). Terms such as “surgery”, “onset of illness” and “hospital admission” were considered equivalent to “ICU admission.”

Assessment time point referred to when assessments were conducted, regardless of reference point. These were classified as ICU discharge, hospital discharge, within 1 month, monthly, or yearly. When only planned timing was described in the methods, it was used as the representative assessment time point. If results included actual timing, that was recorded. Initial and final assessments were also analyzed separately. The reference points for PICS assessments were not taken into account in this analysis; for instance, “1 month after discharge” and “1 month after ICU discharge” were both categorized simply as “1 month.” Additionally, the assessment time points were summarized separately for the initial and final assessments. If a study conducted only a single assessment, the assessment time point was counted in both the initial and final categories.

Frequency was defined as the total number of assessments per study. Intervals between assessments were categorized as within 1 month, monthly, or yearly. In studies with multiple assessments, all intervals were calculated and categorized accordingly.

### Expert survey in the PICS committee of the Japanese Society of Intensive Care Medicine

The expert survey was conducted among all 23 members of the PICS committee and working group of the Japanese Society of Intensive Care Medicine. The survey explored the following: (1) optimal reference point used to determine assessment schedules, (2) optimal assessment time point, (3) optimal frequency of assessment. The questionnaire is shown in Additional File 2. Multiple selections and open-ended responses were allowed. Two rounds of surveys were conducted in August and November 2024. After the first round, an online meeting was held to discuss questionnaire content and clarify its intent. Reminders were sent until all members responded, and full participation was achieved in both rounds.

## Results

**Fig. 1:**
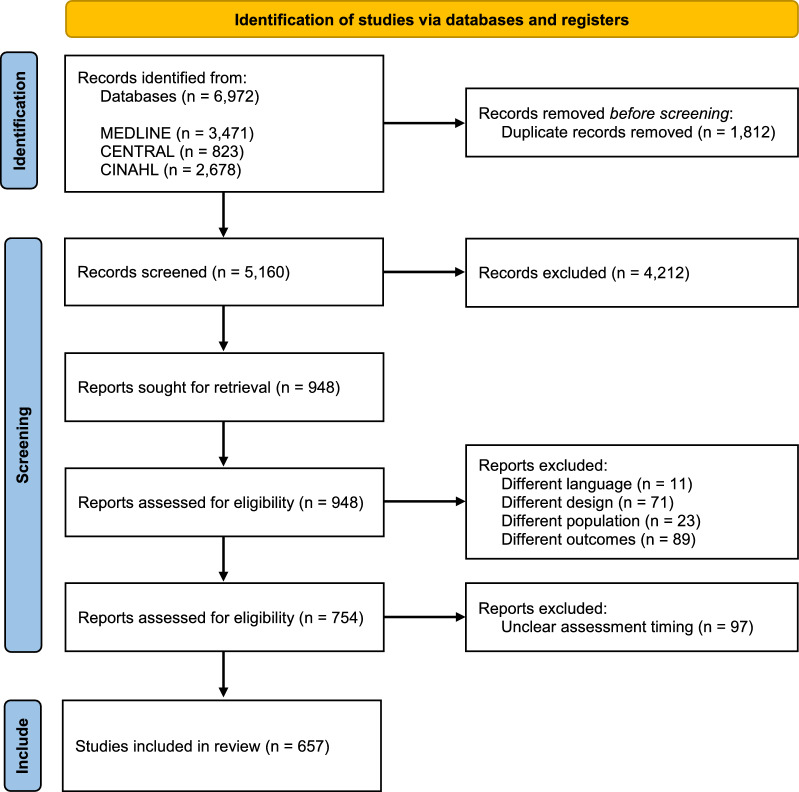
The PRISMA diagram for the scoping review. A total of 948 reports were assessed for eligibility, and 754 reports were included in the original study. Additional screening was carried out on 754 reports, and 657 studies were included in this study

A total of 5,160 studies were screened, and 754 studies were included in the previous review [8]. After further screening for clarity of assessment timing, 657 studies were included in the current analysis (Fig. 1). All included articles are listed in Additional File 3. The number of studies assessing each PICS domain was as follows: physical function (n = 261), cognitive function (n = 133), mental health (n = 242), QOL (n = 273), and PICS-F (n = 69).

**Fig. 2:**
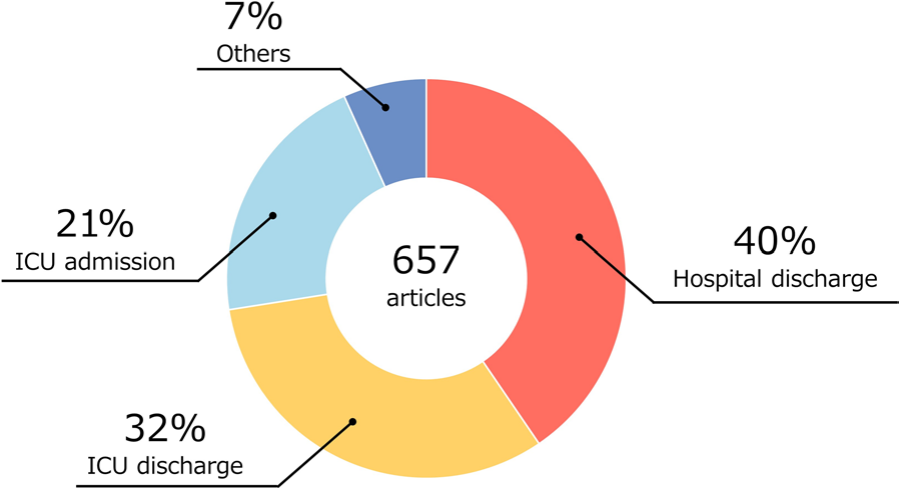
Reference point used to determine assessment schedules in included studies

Figure 2 presents the distribution of reference points used to determine the assessment schedules. Hospital discharge was the most common (n = 240, 40%), followed by ICU discharge (n = 190, 32%), and ICU admission (n = 123, 21%).

**Fig. 3:**
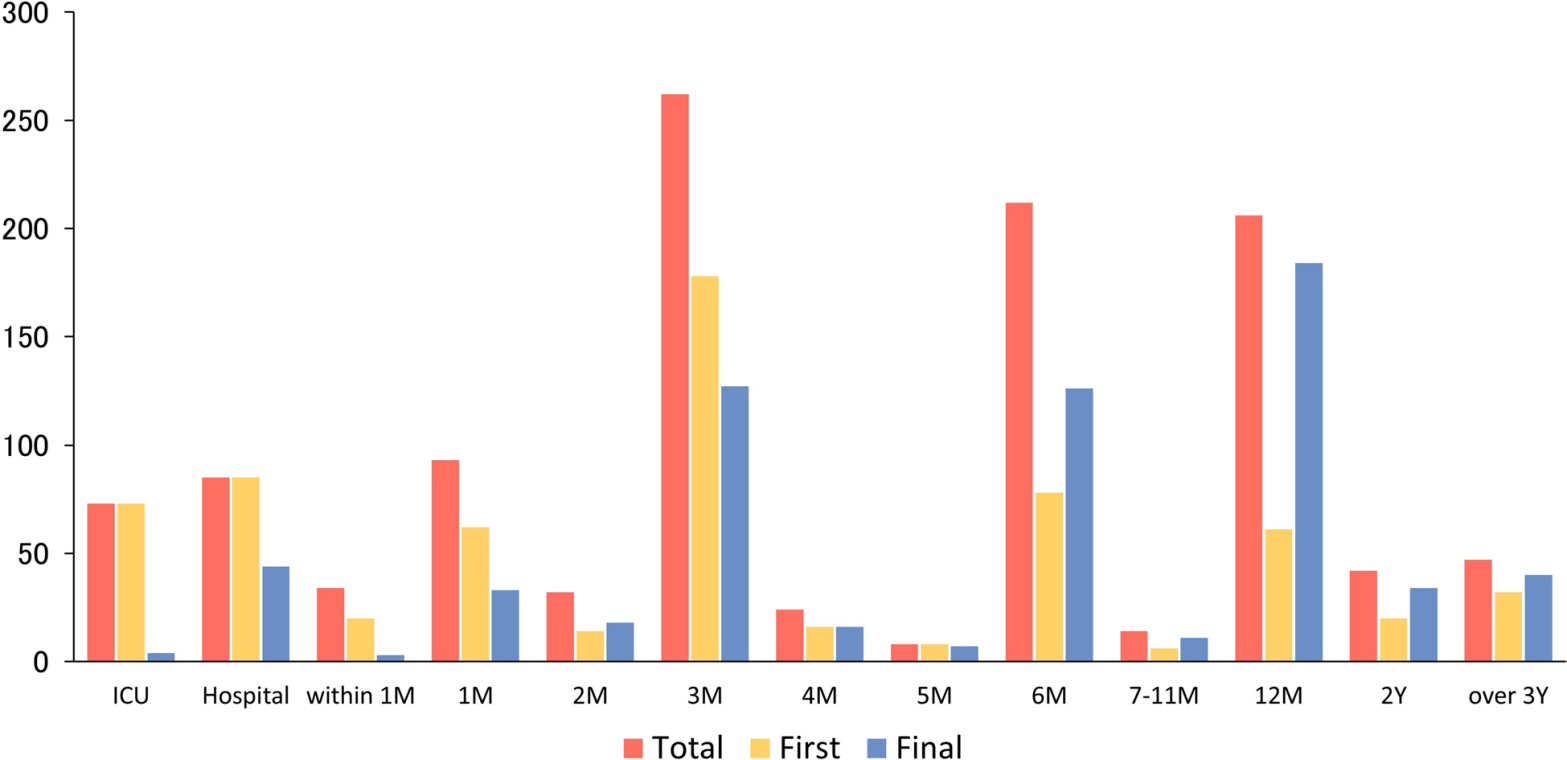
Assessment time point in included studies. Results are shown in the total, first, and final assessments, respectively. If the studies had conducted a single assessment, the assessment time points were counted in both the first and final categories

Figure 3 summarizes the PICS assessment time points. Across all studies, a total of 1,131 assessments were counted. The most frequent time points were 3 months (n = 262, 23%), 6 months (n = 212, 19%), and 12 months (n = 206, 18%). Assessments at ICU and hospital discharge occurred 73 (6%) and 85 (8%) times, respectively. Only 34 (3%) assessments were conducted within 1 month post-discharge, and 89 (8%) occurred more than 1 year post-discharge. The first and final assessments most commonly occurred at 3 and 12 months, respectively. Domain-specific timing data are provided in Additional File 4.

**Fig. 4:**
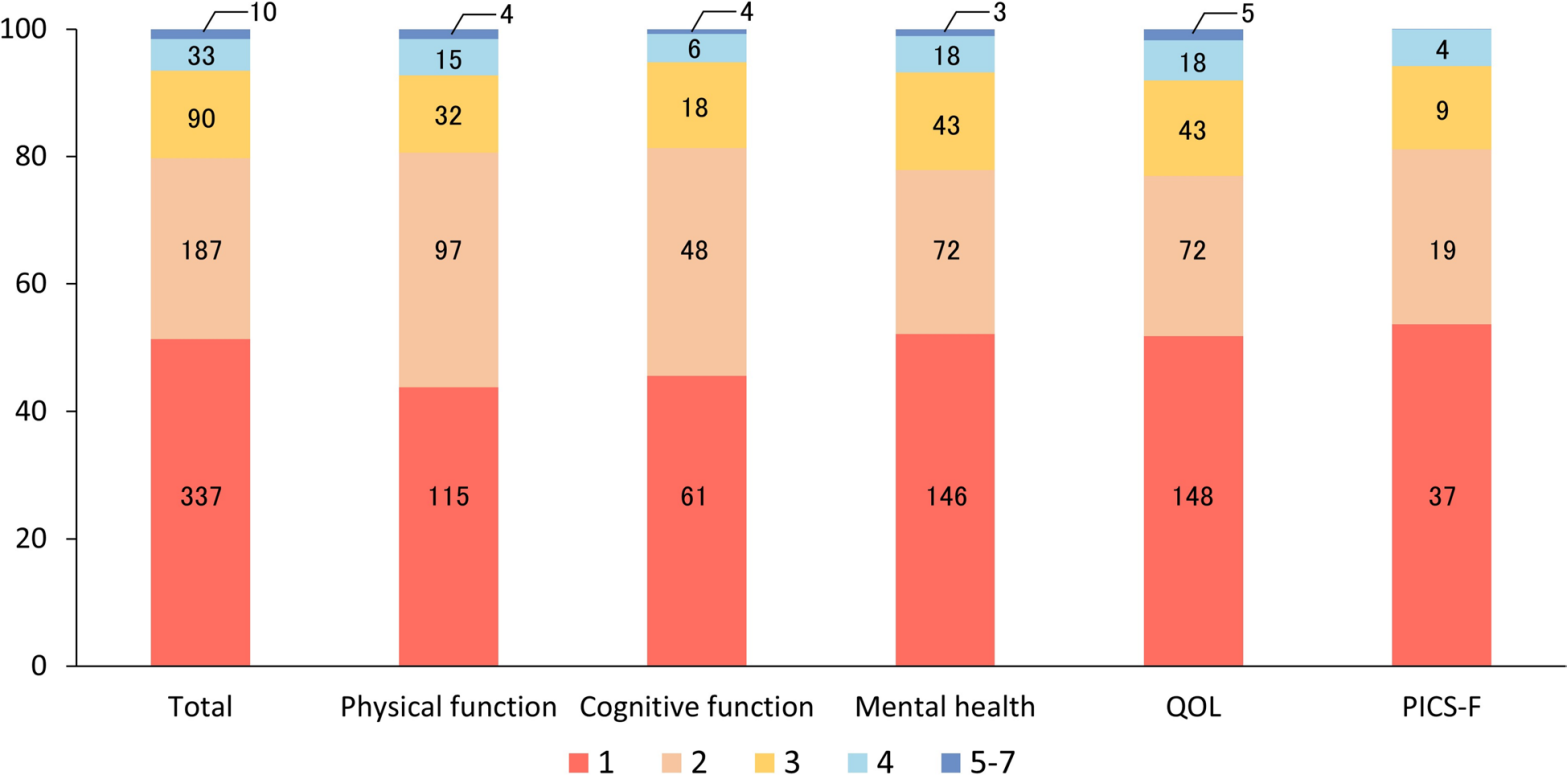
Frequency of assessment in included studies. The results of assessment frequency are shown as percentages. The legends in the figure illustrates the assessment frequency of each color in the bar. The number of assessments conducted in the included studies is presented in the bar chart. QOL: quality of life, PICS-F: post-intensive care syndrome-family

The frequency of PICS assessment is shown in Fig. 4. The most common frequency was a single assessment (n = 337 studies, 51%), followed by two (n = 187, 28%) and three (n = 90, 14%) assessments. Only 43 studies (7%) conducted four or more assessments. Timing and frequency patterns were generally consistent across different PICS domains. Intervals are summarized in Additional File 5, with 3-month (n = 131, 27%) and 6-month (n = 102, 21%) intervals being most common. Studies with two assessment time points most frequently used 6-month intervals, while those with three assessments typically used 3-month intervals.

The participating experts comprised 16 physicians (median years of experience: 19), 3 nurses (22), and 4 physical therapists (32). The survey results are summarized in Table 1. ICU discharge was the most commonly selected reference point (n = 16, 69.6%), followed by hospital discharge (n = 10, 43.5%). The most frequently chosen assessment time point was from 6 to 12 months (n = 12, 52.2%). The optimal frequency was three assessments (n = 12, 52.2%), followed by two assessments (n = 8, 34.8%). The complete response data from the 23 experts are presented in Additional File 6.

**Table 1:** Results of expert survey via online questionnaire

	Results	Advantages	Disadvantages
Reference point for determining assessment schedules			
ICU admission	8 (34.8%)	Starting point can be standardized by single factor that patients need intensive care.	This may not be consistent with the meaning of the term “post-intensive care”.
ICU discharge	16 (69.6%)	This is consistent with the meaning of the term “post-intensive care”.	The length of ICU and hospital stay may depend on various factors such as disease severity, hospital regulations and social issues.
Hospital discharge	10 (43.5%)	The operation of outpatient follow-up may be easy to manage, because assessment date can be conveniently set based on hospital discharge day.	
Time point of assessment			
Within 1 month	7 (30.4%)	The early first assessment facilitates follow-up planning based on severity of symptoms and patient’s needs.	Patients may not discharge from hospital in case of prolonged treatment. (If starting point is ICU admission or ICU discharge.)
More than 1 and less than 3 months	10 (43.5%)	The problems that patients have in their home life after hospital discharge can be assessed.	
More than 3 and less than 6 months	11 (47.8%)	Symptoms may be stable in some patients, while others may have recurrence or remission. And thus, long-term assessment enables to observe changes in symptoms.	The assessment date is set at a long interval after hospital discharge, which risks failing to assess the problems patients already have. (If this is the first assessment.).
More than 6 and less than 12 months	12 (52.2%)		
More than 12 and less than 24 months	11 (47.8%)		
More than 24 months	6 (26.1%)		
Frequency of assessment			
One time	3 (13.0%)	Less burden on patients and family members as it is completed in a single assessment.	Single assessment cannot observe chronological changes in symptoms.
Two times	8 (34.8%)	Repeated assessments enable observation of changes in symptoms.	Repeated assessments are burdensome for patients and family members, making it difficult to continue follow-up.
Three times	12 (52.2%)		
Four times	3 (13.0%)		
More than five times	1 (4.3%)		

Multiple selections were allowed in the expert survey

## Discussion

This study revealed discrepancies between practices in previous studies and expert recommendations regarding PICS assessment timing. Most prior studies used hospital discharge as the reference point, whereas experts preferred ICU discharge, aligning with the concept of “post-intensive care” and enabling the tracking of patients. Regarding the assessment time points, previous studies commonly set them at 3, 6, and 12 months. PICS experts also indicated that assessments would be better conducted between 6 and 12 months. While the optimal frequency of PICS assessments according to experts was three times in total, most previous studies conducted only a single assessment.

The reference point at ICU discharge has the advantage of clarifying how long symptoms persist following ICU discharge, aligning with the “post-intensive care syndrome” definition. On the other hand, the reference point at hospital discharge is beneficial to determine the follow-up schedule after hospital discharge. However, the use of ICU or hospital discharge as a reference point has a disadvantage because the length of ICU or hospital stay is different among patients. The length is affected by various factors such as patients’ background, treatment, and hospital policies [12–15]. In contrast, reference point at ICU admission is determined by a single criterion that the patient required intensive care. Given these influences, ICU admission as a reference point has the advantage of being a more objective and uniform reference point among different patients.

Regarding assessment time points, there was a notable similarity between previous studies and the expert survey results at 6 and 12 months post-discharge. To our knowledge, no prior studies have specifically examined the rationale for selecting assessment time points for PICS, and it was based on precedent. On the other hand, the Society of Critical Care Medicine recommends screening PICS-related problems within 1 month after discharge [11]. The discrepancy from our results reflects differing objectives. The Society of Critical Care Medicine guidance is intended for clinical practice, emphasizing early screening and follow-up, whereas our study focused on the methodological aspects of clinical research. In clinical research, assessments should be scheduled to observe the longitudinal trend. There is longitudinal fluctuation of PICS symptoms. Physical function tends to improve over time, with notable recovery in mobility and muscle strength over 2 years [16, 17]. The most substantial improvements appear to occur within the first 6 months after discharge [18–21]. In contrast, while some studies have reported short-term improvements in cognitive function and mental health [22–24], these impairments often persist in the long term [25]. Notably, patients with moderate to severe cognitive impairment showed minimal change over several years [26]. Further, mental health symptoms showed patterns of remission and relapse during 2-year follow-up periods [7]. Regarding PICS-F, prevalence rates declined during the first 6 months but remained relatively stable thereafter [27]. Taken together, these findings suggest that symptom stabilization is observed after 6 months following discharge. Therefore, to capture a reliable picture of post-ICU sequelae, it may be optimal to conduct assessment between 6 and 12 months after discharge.

The most common response among the experts was that PICS should be assessed three times, whereas previous studies most frequently employed a single assessment. This difference may be attributed to the increased workload associated with frequent assessments, which can impact the feasibility of conducting studies. Furthermore, increasing the number of assessments also raises the risk of patient dropout. In fact, studies that conducted multiple assessments reported dropouts at each follow-up point [28]. Although three assessments were considered the optimal frequency by experts, the associated workload and potential dropout rates should be carefully considered.

This study has several limitations. First, this scoping review and expert survey cannot determine the optimal timing of PICS assessments in clinical practice, as we focused on clinical research. Second, the questionnaire options for assessment time points were categorized rather broadly, as it is difficult to choose the detailed options for months. Similarly, the optimal time point and frequency of assessment for each PICS domain were not addressed in our expert survey. Further studies are necessary to determine the optimal timing of assessments for each PICS domain. Furthermore, this study’s expert survey was limited to a small sample of 23 Japanese experts. Given that approaches to and perceptions of PICS may vary depending on healthcare systems, the generalizability of the findings to other international settings may be limited. Accordingly, the results should be interpreted with caution, taking into account the regional context. Finally, the assessment time points did not account for the reference point (e.g., ICU admission, ICU discharge, or hospital discharge). As a result, the assessment time points may vary due to the different ICU or hospital length of stay.

## Conclusions

We identified differences between research practices and expert opinions regarding the timing of PICS assessments through a scoping review and expert survey. Further research of optimal timing is needed to reduce methodological heterogeneity in future PICS research.

## Supplementary Information

Additional file 1. The search strategies for the original scoping review.

Additional file 2. The contents of questionnaire for the expert survey.

Additional file 3. All article list included in this study.

Additional file 4. The assessment time point in each PICS domain. A: results of total assessments, B: time points for initial assessment, C: time points for final assessment. QOL: quality of life, PICS-F: post-intensive care syndrome-family.

Additional file 5. Interval between each PICS assessment in included studies. A: results of total assessments, B: results of the each assessment frequency. The legends in the figure illustrates the assessment frequency of each color in the bar.

Additional file 6. The complete response data of the expert survey.

## Data Availability

No datasets were generated or analysed during the current study.

## Abbreviations

ICU: Intensive care unit
PICS: Post-intensive care syndrome
PICS-F: Post-intensive care syndrome-family
QOL: Quality of life
