# Supplementary material for: Optimal timing for assessing post-intensive care syndrome in clinical research: a scoping review and expert survey
Source: J Intensive Care. 2025 Aug 18;13:45. doi: 10.1186/s40560-025-00817-8 (PMC12359912; doi:10.1186/s40560-025-00817-8)
Supplement: Supplementary file 1 — Additional file 1. The search strategies for the original scoping review. [file 40560_2025_817_MOESM1_ESM.docx]

**Search strategy**

**Databases**

- MEDLINE
- CENTRAL
- CINAHL

Among 6972 identified records, 5160 were included after the deletion of duplicates.

MEDLINE (via PubMed) search strategy (Searched in AM–08:10, November 27, 2022)

|  | Search formula | Results |
| --- | --- | --- |
| #1 | (intensive care[tiab] OR ICU[tiab] OR ICUs[tiab] OR "intensive care units"[MeSH Terms] OR intensive therapy[tiab] OR critical care[tiab] OR "critical care"[MeSH Terms]) AND (survivors[tiab] OR postintensive care syndrome[tiab] OR post intensive care syndrome[tiab] OR PICS[tiab] OR family[tiab]) AND (impairment[tiab] OR physical[tiab] OR cognitive[tiab] OR mental[tiab] OR posttraumatic stress disorder[tiab] OR depression[tiab] OR anxiety[tiab] OR “activities of daily living"[MeSH Terms] OR ADL[tiab] OR quality of life[tiab] OR "quality of life"[MeSH Terms] OR morbidity[tiab] OR "morbidity"[MeSH Terms]) NOT (animals[mh] NOT humans[mh]) | 5835 |
| #2 | Limits: 2014/Jan/1 – present | 3471 |

Cochrane Central Register of Controlled Trials search strategy (Searched in A–08:46, November 27, 2022)

| #1 | ("intensive care"):ti,ab,kw | 27771 |
| --- | --- | --- |
| #2 | ("ICU"):ti,ab,kw | 16086 |
| #3 | ("ICUs"):ti,ab,kw | 1744 |
| #4 | MeSH descriptor: [Intensive Care Units] explode all trees | 4145 |
| #5 | ("intensive therapy"):ti,ab,kw | 1061 |
| #6 | ("critical care"):ti,ab,kw | 4519 |
| #7 | MeSH descriptor: [Critical Care] explode all trees | 2238 |
| #8 | #1 OR #2 OR #3 OR #4 OR #5 OR #6 OR #7 | 37647 |
| #9 | ("survivors"):ti,ab,kw | 12622 |
| #10 | ("postintensive care syndrome"):ti,ab,kw | 54 |
| #11 | ("post intensive care syndrome*"):ti,ab,kw | 70 |
| #12 | ("PICS"):ti,ab,kw | 117 |
| #13 | ("family"):ti,ab,kw | 39219 |
| #14 | #9 OR #10 OR #11 OR #12 OR #13 | 51355 |
| #15 | ("impairment"):ti,ab,kw | 38293 |
| #16 | ("physical"):ti,ab,kw | 142628 |
| #17 | ("cognitive"):ti,ab,kw | 84978 |
| #18 | ("mental"):ti,ab,kw | 70589 |
| #19 | ("posttraumatic stress disorder"):ti,ab,kw | 5517 |
| #20 | (“depression”):ti,ab,kw | 90609 |
| #21 | ("anxiety"):ti,ab,kw | 64582 |
| #22 | MeSH descriptor: [Activities of Daily Living] explode all trees | 10374 |
| #23 | ("ADL"):ti,ab,kw | 4159 |
| #24 | ("quality of life"):ti,ab,kw | 135560 |
| #25 | MeSH descriptor: [Quality of Life] explode all trees | 29546 |
| #26 | ("morbidity"):ti,ab,kw | 43154 |
| #27 | MeSH descriptor: [Morbidity] explode all trees | 15984 |
| #28 | #15 OR #16 OR #17 OR #18 OR #19 OR #20 OR #21 OR #22 OR #23 OR #24 OR #25 OR #26 OR #27 | 471247 |
| #32 | #8 AND #14 AND #28 | 1112 |
| #33 | Limits: trial | 1087 |
| #34 | Limits: 2014/Jan/1 – present | 823 |

CINAHL (Searched in AM–9:17, November 27, 2022)

| #1 | ("intensive care" OR "ICU" OR "ICUs" OR MH "intensive care units" OR "intensive therapy" OR "critical care" OR MH "critical care") AND ("survivors" OR "postintensive care syndrome" OR "post intensive care syndrome" OR "PICS" OR "family") AND ("impairment" OR "physical" OR "cognitive" OR "mental" OR "posttraumatic stress disorder" OR "depression" OR "anxiety" OR MH "activities of daily living" OR "ADL" OR "quality of life" OR MH "quality of life" OR "morbidity" OR MH "morbidity") | 4315 |
| --- | --- | --- |
| #2 | Limits: 2014/Jan/1 – present | 2678 |
