# Supplementary material for: Optimal timing for assessing post-intensive care syndrome in clinical research: a scoping review and expert survey
Source: J Intensive Care. 2025 Aug 18;13:45. doi: 10.1186/s40560-025-00817-8 (PMC12359912; doi:10.1186/s40560-025-00817-8)
Supplement: Supplementary file 2 — Additional file 2. The contents of questionnaire for the expert survey. [file 40560_2025_817_MOESM2_ESM.docx]

Additional file 3. The contents of questionnaire for the expert survey

| **Q1. Reference point for determining assessment schedules** |  |
| --- | --- |
| Q1-1. When is the best starting point for counting PICS assessment date?  Please select the option you think is appropriate. (Multiple answers allowed) | 1. ICU admission |
|  | 2. ICU discharge |
|  | 3. Hospital discharge |
| Q1-2. Please write any comments you have regarding the above question. |  |
| **Q2. Time point of assessment** |  |
| Q2-1. When is the best timing for PICS assessment?  Please select the option you think is appropriate. (Multiple answers allowed) | 1. Within 1 month |
|  | 2. More than 1 and less than 3 months |
|  | 3. More than 3 and less than 6 months |
|  | 4. More than 6 and less than 12 months |
|  | 5. More than 12 and less than 24 months |
|  | 6. More than 24 months |
| Q2-2. Please write any comments you have regarding the above question. |  |
| **Q3. Frequency of assessment** |  |
| Q3-1. How many times are the best frequency for PICS assessment?  Please select the option you think is appropriate. (Multiple answers allowed) | 1. One time |
|  | 2. Two times |
|  | 3. Three times |
|  | 4. Four times |
|  | 5. More than five times |
| Q3-2. Please write any comments you have regarding the above question. |  |
