# Supplementary figures and images for: Optimal timing for assessing post-intensive care syndrome in clinical research: a scoping review and expert survey
Source: J Intensive Care. 2025 Aug 18;13:45. doi: 10.1186/s40560-025-00817-8 (PMC12359912; doi:10.1186/s40560-025-00817-8)

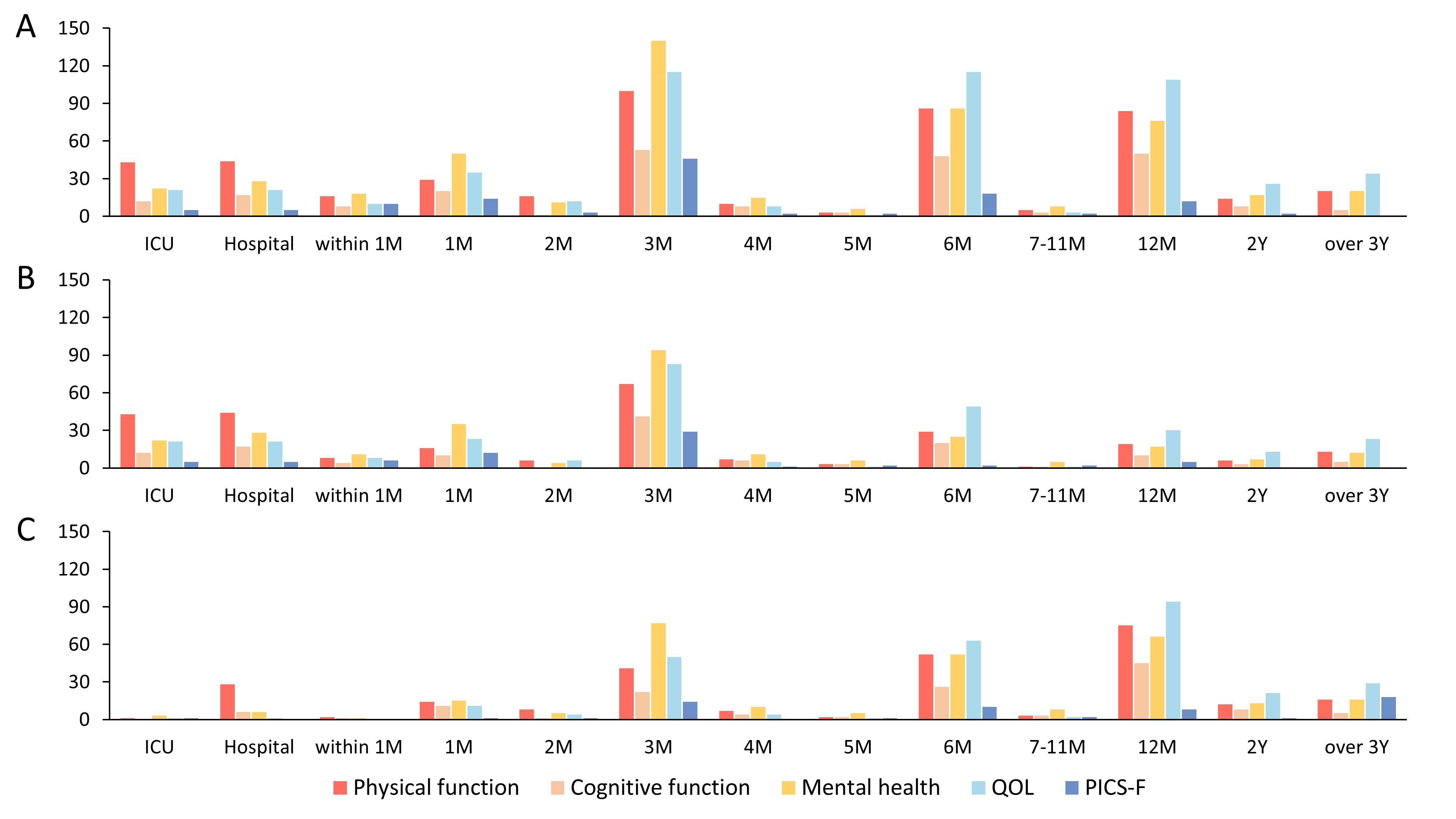

Supplement: Supplementary file 4 — Additional file 4. The assessment time point in each PICS domain. A: results of total assessments, B: time points for initial assessment, C: time points for final assessment. QOL: quality of life, PICS-F: post-intensive care syndrome-family. [file 40560_2025_817_MOESM4_ESM.jpg]

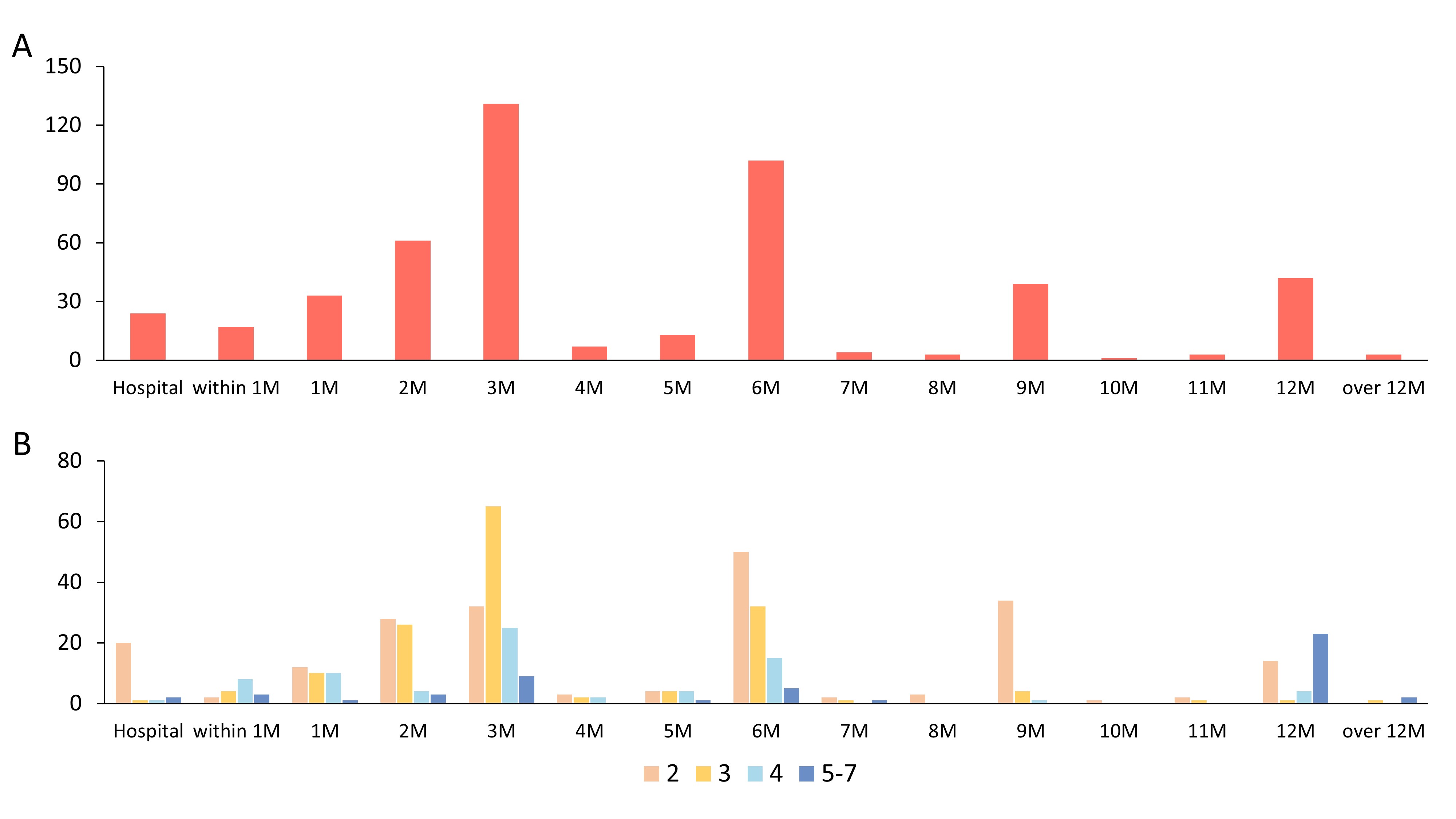

Supplement: Supplementary file 5 — Additional file 5. Interval between each PICS assessment in included studies. A: results of total assessments, B: results of the each assessment frequency. The legends in the figure illustrates the assessment frequency of each color in the bar. [file 40560_2025_817_MOESM5_ESM.jpg]
